# Correction: John F. R. Kerr (1934–2024)

**DOI:** 10.1038/s41418-024-01373-8

**Published:** 2024-09-12

**Authors:** Margaret C. Cummings, David L. Vaux, Andreas Strasser, Ruth Kluck

**Affiliations:** 1 Pathology Queensland, Royal Brisbane and Women’s Hospital, Brisbane, QLD Australia; 2 Centre for Clinical Research, Faculty of Medicine, The University of Queensland, Brisbane, QLD Australia; 3 The Walter and Eliza Hall Institute of Medical Research, Melbourne, VIC Australia; 4 Department of Medical Biology, University of Melbourne, Melbourne, VIC Australia

Correction to: *Cell Death & Differentiation* 10.1038/s41418-024-01338-x, published online 29 July 2024

The article “John F. R. Kerr (1934–2024)”, written by Margaret C. Cummings, David L. Vaux, Andreas Strasser and Ruth Kluck was originally published online on the publisher’s internet portal on 29th of July 2024 with Open Access under a Creative Commons Attribution (CC BY) license 4.0.

With the author’s/authors’ decision to cancel Open Access the copyright of the article changed on 16 August 2024 to “© The Author(s), under exclusive licence to ADMC Associazione Differenziamento e Morte Cellulare 2024” with all rights reserved.

The original article has been corrected

